# Acute carpal tunnel syndrome due to a hemangioma of the median nerve

**DOI:** 10.4103/0019-5413.30532

**Published:** 2007

**Authors:** DS Meena, Mrinal Sharma, CS Sharma, Purnima Patni

**Affiliations:** Dept. of Orthopedics, SMS Hospital, Jaipur, India

**Keywords:** Acute Carpal tunnel syndrome, hemangioma, median nerve

## Abstract

Hemangioma of the median nerve presenting as acute carpal tunnel syndrome is unusual A-18- year old male presented with severe incapacitating pain of sudden onset of left forearm and hand after manual field work. There was swelling on volar aspect of forearm, with hyperalgesia in the median nerve distribution. The fingers and wrist were inmarked flexion and the patient did not allow wrist and finger extension. X-rays were within normal limits. An emergency volar carpal ligament release revealed, haematoma about 100 ml with numerous vessels encircling the median nerve. Histopathology of lesion turned out to be a cavernous hemangioma. Post operatively patient had full recovery.

Chronic carpal tunnel syndrome (CTS) is a unique entity in that it is the most common entrapment neuropathy encountered in neurosurgical practice. A thorough search of the literature has revealed that till now two cases of chronic CTS due to hemangioma involving the neurovascular structures in the wrist and forearm have been reported.

We are reporting here a case of hemangioma of the median nerve with a presentation of acute CTS.

## CASE REPORT

**Figure 1:**
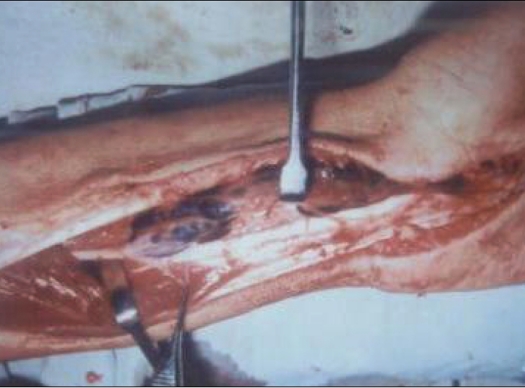
Intraoperative photograph showing decompression by volar incision

In May 1996, a young manual laborer of 18 years presented with agonizing pain in his left forearm and hand for the past two days. Patient developed this pain after having exerted during manual fieldwork. Pain was sudden in onset, severe and incapacitating, not responding to analgesics. A diffuse, nonreducible swelling was present on the volar aspect of the forearm [Figure 1]. No pulsations were present, local temperature was raised. Fingers and wrist were in marked flexion and the patient did not allow wrist and finger extension. There was hyperalgesia in the median nerve distribution. There was no wasting of the thenar muscles or history of paresthesias at night suggestive of a chronic cause. Regional lymph nodes were not enlarged. Routine investigations and chest X-ray were normal. An MRI could not be performed as the facilities were not available at that time. Carpal pillars projection showed no bony abnormality. An emergency surgery was done under tourniquet and volar carpal ligament was released. Intraoperative findings showed hematoma about 100cc along with numerous vessels encircling the median nerve and intermingled within the substance of the median nerve. No definite plane of cleavage could be found and we were not able to resect the lesion and the mass was left inside after taking a microbiopsy.

Histopathology proved it to be a cavernous hemangioma. Postoperatively patient showed full recovery of functions with normal sensations although he had to change his job from a manual worker to a vendor.

## DISCUSSION

CTS (tardy median nerve palsy) described in 1854 by Sir James Paget, is the most common entrapment neuropathy encountered in neurosurgical practice. Most cases are thought to be idiopathic in nature, presumably resulting from cumulative repetitive microtrauma in individuals with congenitally small carpal canals. It occurs in middle-aged obese women. It is usually bilateral and may occur due to increase in canal contents, decreased canal size, neuropathic inflammatory conditions and conditions due to altered fluid balance. Hamartomas involving the peripheral nerves were first described in 1953 by Mason,^1^ mostly involving the median nerve in the forearm and wrist and usually presenting as chronic CTS. Acute CTS may occur consecutive to hamate and triquetral fractures,^2^ acute metacarpal osteomyelitis^3^ compression by anomalous flexor digitorum superficialis,^4^ after internal fixation of scaphoid,^4^ secondary to pyogenic infections,^5^ classical hemophilia,^6^ von Willebrand's disease^7^ oral anticoagulant therapy,^8^ distal radial fractures,^9^ filarial infections,^10^ thrombosed persistent median artery,^11^ idiopathic tumoral calcinosis,^12^ decompression sickness,^13^ Hansen's disease,^14^ peritendinitis calcarea,^15^ pseudogout,^16^ scaphoid pseudoarthroses.^17^ Bilateral cases may occur due to human parvovirus B19 infection.^18^

Pathophysiology of carpal tunnel is typically demyelination. Tenosynovitis is not a part of the pathophysiological process in chronic idiopathic CTS. Acute CTS may be thought of as a compartment syndrome of carpal canal and decompression should be performed as early as possible. Acute CTS can be diagnosed through history and physical examination alone and electrophysiological studies are not required. Acute CTS differs from chronic CTS in that the presentation is of sudden onset and characteristic features of chronic CTS such as thenar wasting, trophic changes, night pain and paresthesias in median nerve distribution may not be present.

To the best of our knowledge only two cases of chronic CTS due to a hemangioma involving the neurovascular structures in the wrist and forearm have been reported. Although 44 cases of fibrolipomatous hamartomas involving peripheral nerves have been reported, nearly half of them by Silverman and Enzinger,^19^ 41 involved the median nerve, two in the ulnar nerve,^20^ one in an unidentified nerve on the extensor surface of the proximal forearm and one in a case of macrodactyly.^21^ Patel *et al*^22^ have reported two cases of intraneural hemangioma producing compression of the median nerve and requiring multiple excisions. In one case, intrafascicular nerve dissection failed to produce a cure, while the other patient remained free of recurrence after excision of the involved median nerve and sural nerve grafting. Peled *et al*^23^ reported a case of an extensive cavernous hemangioma with presentation of chronic CTS. Murali *et al*^24^ reported a case of lipofibromatous hamartoma of the median nerve presenting with CTS of six months duration in a 63-year-old woman. Our case differs from the others reported so far in that the patient is an adolescent male who had an acute presentation of CTS and the mass was left *in situ* as no plane of cleavage could be found. In our ten year follow-up he has had no recurrence of symptoms or thenar wasting till date although he had to change his profession from a manual worker to a vendor to avoid recurrence of symptoms due to traumatic rupture of vessels in the hemangioma.

Whenever a patient presents with s/s of acute CTS the possibility of a hemangioma involving the median nerve should be kept in mind in the differential diagnosis. Surgery can be limited to decompression only if a plane of cleavage cannot be found. If symptoms recur or progress later, total resection and grafting may then be carried out, although the results are not satisfactory^22^
